# ﻿A new barracudina (Aulopiformes, Paralepididae, *Stemonosudis*) from Somalia, with additional records of *S.siliquiventer* from the Caribbean Sea

**DOI:** 10.3897/zookeys.1241.138677

**Published:** 2025-06-12

**Authors:** Hsuan-Ching Ho, Tsung-Yu Yang

**Affiliations:** 1 Department and Graduate Institute of Aquaculture, National Kaohsiung University of Science and Technology, Kaohsiung, Taiwan; 2 Taiwan Ocean Research Institute, National Institutes of Applied Research, Kaohsiung, Taiwan; 3 Australian Museum, Sydney, Australia; 4 National Museum of Marine Biology & Aquarium, Pingtung, Taiwan; 5 Department of Fisheries Technology and Management, National Kaohsiung University of Science and Technology, Kaohsiung, Taiwan

**Keywords:** Biodiversity, ichthyofauna, ichthyology, Lestidiinae, taxonomy

## Abstract

A new species, *Stemonosudisadenensis***sp. nov.**, of slender barracudina is described based on a specimen collected off Somalia, Western Indian Ocean. The new species differs from its congeners by having a uniformly brown body, without distinct black patches; nostrils slightly before the posterior end of the maxilla; anus above the tip of the appressed pelvic fin; lateral-line scales: prepelvic 39, predorsal 48, preanal 59, total 81; vertebrae: prepelvic 38, prehaemal 42, predorsal 46, preanal 59, caudal 53, and total 95; and total gill rakers 68. Additional records of *Stemonosudissiliquiventer* Post, 1970 from the Caribbean Sea are also provided.

## ﻿Introduction

The barracudina genus *Stemonosudis* Harry, 1951 comprises a group of relatively to extremely slender fishes inhabiting the Pacific, Atlantic and Indian oceans. There are approximately 14 nominal species, most of which were originally described from juveniles (Ege 1957; Rofen 1966; Ho et al. 2019a, b). Some species still lack adult information, but those of some species have been described in detail recently (Ho et al. 2019a; Ho and Huang 2022).

Rofen (1966: 422) included 10 species of *Stemonosudis* in his key to the species. Richards (1967) described *S.rothschildi* Richards, 1967 from the central Pacific Ocean, and Post (1970) described *S.siliquiventer* Post, 1970 from the western central Atlantic Ocean. Nearly fifty years later, Ho et al. (2019a) provided data and descriptions of four *Stemonosudis* species from Taiwan; Ho et al. (2019b) reviewed the *S.rothschildi* species complex and described two new species, *S.multifasciata* Ho, Russell, Graham & Psomadakis, 2019 and *S.retrodorsalis* Ho, Russell, Graham & Psomadakis, 2019; and Ho and Huang (2022) reported the adults of *Stemonosudiselongata* (Ege, 1933) having a uniformly black body.

As part of a broader effort investigating all known paralepidids, we examined specimens primarily based on collections of the National Museum of Natural History, Washington, D.C. and Museum of Comparative Zoology, Boston. Among these, we identified a unique specimen collected from off Somalia, which has a relative short body, uniformly brown coloration, and several distinct characteristics. We describe this specimen as a new species and compare it with similar congeners. Additionally, *Stemonosudissiliquiventer*, originally described from the western central Atlantic, is redescribed based on five specimens collected from the Caribbean Sea.

## ﻿Material and methods

Standard length (SL) and head length (HL) are used throughout. Methods for measurements and counts follow Ho et al. (2019a) and Ho and Lin (2023), with an additional measurement of pre-anus length, measured from the tip of the snout to the genital papilla. Specimens examined are deposited at the National Museum of Natural History, Smithsonian Institution (**USNM**) and Museum of Comparative Zoology, Harvard University (**MCZ**). Vertebral counts were made from radiographs taken by a DURASCAN 1417 digital x-ray system at USNM. Color photographs were taken using a Nikon D850 and AF-S Micro NIKKOR 60 mm f/2.8G ED lens.

### ﻿Abbreviations

**DFO** = dorsal-fin origin; **VFO** = pelvic-fin origin; **AFO** = anal-fin origin. V–D, the space or distance between VFO and DFO, calculated by predorsal length minus preanal length; **V–A**, the space or distance between VFO and AFO, calculated by preanal length minus prepelvic length. It is notable that the preanal length in Ege (1930, 1933, 1953, 1957) is the length from snout tip to anus, which is equal to “pre-anus length” in this study. **PVLL**, **PDLL**, **PALL** are the numbers of lateral-line scales before VFO, DFO, AFO, respectively; and **TLL** is the total lateral-line scales, including some small scales on the posterior portion of the lateral line. **PVV**, **PDV** and **PAV** are the numbers of vertebrae before VFO, DFO and AFO, respectively; **PHV**, **CV** and **TV** are numbers of prehaemal, caudal and total vertebrae, respectively.

Comparative data were taken from Ege (1933), Rofen (1966), Ho et al. (2019b), Ho and Huang (2022) and examination of specimens (Ho, pers. data).

## ﻿Results

### ﻿Family Paralepididae

#### ﻿ Stemonosudis adenensis

Taxon classificationAnimaliaAulopiformesParalepididae

Ho sp. nov.

6257AAB8-D48B-5B8A-B423-9D850727B2EA

https://zoobank.org/7E902434-0101-4EF2-AC32-76FBCE9D5C18

**Figure 1.**
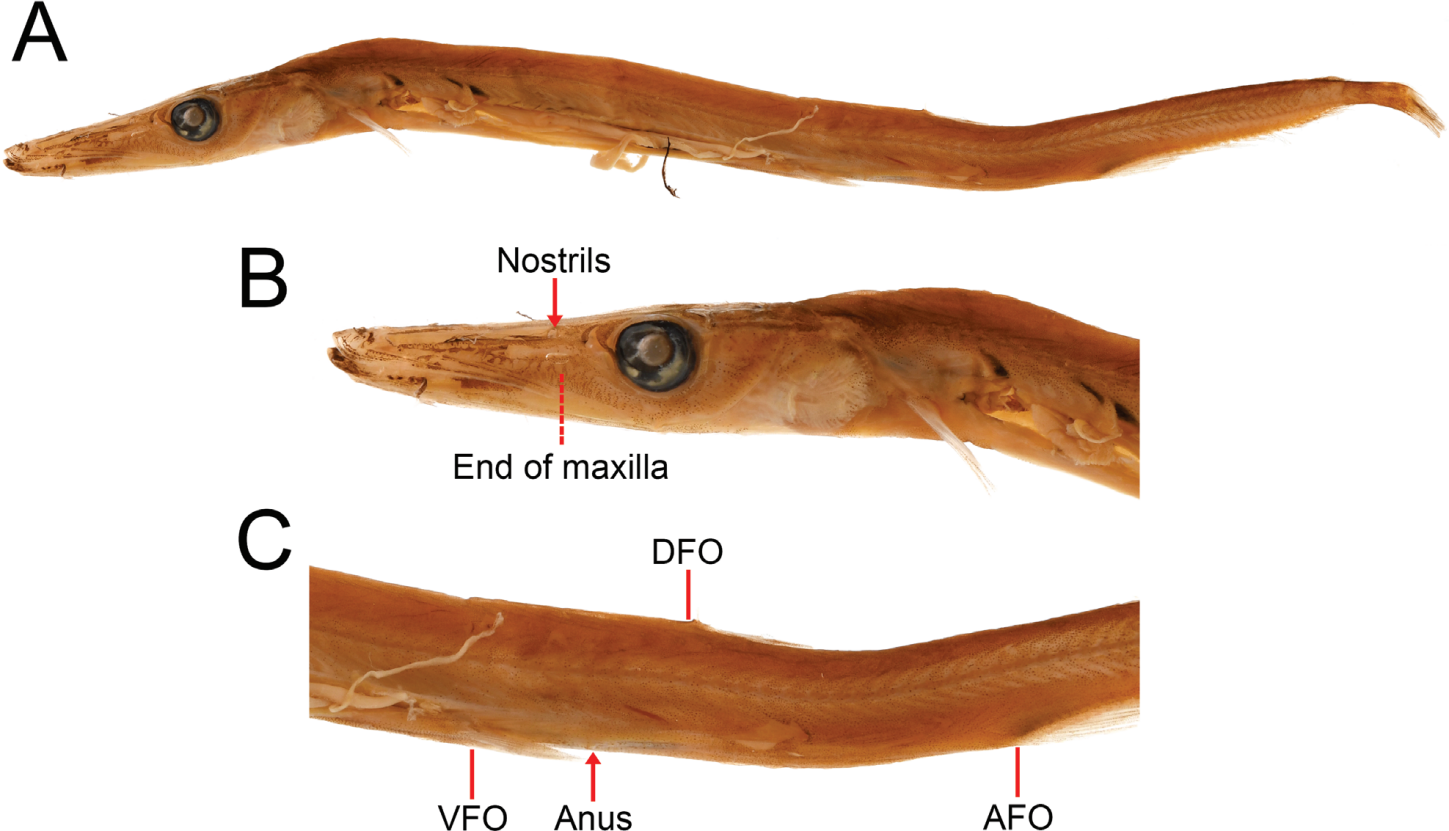
*Stemonosudisadenensis* sp. nov., USNM306145, 186 mm SL**A** lateral view **B** lateral view of head, arrow points to the nostrils and bar indicates the posterior end of maxilla **C** lateral view of trunk, arrow points to the anus and bars indicate origins of pelvic fin (VFO), dorsal fin (DFO) and anal fin (AFO).

Fig. 1A–C , Tables 1 , 2 , 3 Common name: Brown Slender Barracudina

##### Holotype.

• USNM 306145 (186 mm SL), *R/V BEINTA*, cr. 14, haul 13, 11°15'35"N, 48°05'47"E, off Somalia, Gulf of Aden, Western Indian Ocean, 375 m, 15 Sep. 1986, coll. J. June.

##### Diagnosis.

A species of *Stemonosudis* with the following combination of characters: body brown with dorsum slightly darker, without black patches; nostrils slightly before vertical through posterior end of maxilla; anus above tip of appressed pelvic fin; lateral-line scales: PVLL 39, PDLL 48, PALL 59, TLL 81; vertebrae: PVV 38, PHV 42, PDV 46, PAV 59, CV 53, and TV 95; and total gill rakers 68.

##### Description.

Body slender and compressed, relatively short compared to most congeners. Caudal peduncle short, its length ca. 1.2 times eye diameter. Ventral adipose fin barely developed along abdominal ridge before pelvic fin, but well-developed along ventral margin between anus and AFO. Anus located above tip of appressed pelvic fin, well ahead of DFO by about base length of dorsal fin.

Head relatively long, its length 4.2 in SL; snout moderately long and pointed, its length 2.0 in HL or 8.2 in SL. Mouth terminal, moderately large, its gape extends to about one eye diameter before eye when mouth opened; tip of lower jaw slightly upturned, with small fleshy tissue at tip. Eye moderately large, its diameter 6.4 in HL. Interorbital space moderately broad, its width 13.6 in HL; some straight ridges on top of head and snout. Posterior end of maxilla extending to about 2/3 eye diameter in front of the eye, upper-jaw length 2.4 in HL. A skin fold originating from about middle of snout and ending near a vertical through anterior margin of eye. Two nostrils located slightly before a vertical through posterior end of maxilla, situated at about 2/3 eye diameter in front of eye, pre-nostril length 2.5 in HL. Numerous sensory canals on snout, cheek, operculum, and upper jaw; irregular rows of small pores on lower surface of lower jaw.

Gill filaments present on all four gill arches. Fourth arch mostly connected to inner surface of gill chamber by membranes. Pseudobranchs present, anterior half inside a deep pocket.

Dorsal fin small with short base, base length 6.9 in HL. DFO well behind pelvic fin and middle of the fish, well before middle of V–A, predorsal length 1.5 in SL. Pectoral-fin base slightly behind a vertical through posterior margin of gill cover, its upper base about same level of lower margin of eye, pectoral-fin length 2.9 in HL. A small pocket behind pectoral-fin base. Pelvic fin slightly behind middle of body, prepelvic length 1.8 in SL; a small, slender axial scale behind pelvic-fin base. Anal fin with a long base, originating on posterior fourth of body, base length 1.4 in HL. Adipose fin small, its base slightly smaller than eye diameter, situated above rear portion of anal-fin base.

Two small fangs at tip of upper jaw, followed by single row of numerous small retrorse teeth along upper jaw, gradually smaller posteriorly. Vomerine teeth absent. Two fangs at front of lower jaw, followed by 2 rows of fangs on lower jaw, forming about 7 tooth pairs, those in inner row distinctly long, depressible, with a knife-like tip; those in outer row much shorter, retrorse and fixed. Two rows of fangs on each palatine, anterior teeth forming 5 (right) or 7 (left) widely-spaced tooth pairs, those in inner row depressible and distinctly longer than those on outer row, small and fixed; posterior portion with single row of small fixed teeth. Two long rows of small retrorse teeth on tongue.

Gill rakers present on all gill arches; small, shield-shaped, usually with 3–5 small teeth and a narrow base, teeth not especially emergent over margin of gill arch. Teeth on pharyngeal arch slender, forming an oval patch with about 4 (4–5) rows at middle. Single row of small teeth on fifth ceratobranchials, forming a V-shaped pattern.

Body scaleless, except for a single row of lateral-line scales originating above pectoral girdle and extending to a vertical through about 2/3 of length of anal-fin base. Lateral-line scales relatively long, longer than height, gradually smaller and becoming narrower posteriorly; single row of 3 large pores on upper and lower margin of each scale, middle pore smaller. Luminescent duct absent.

***Coloration*.** When preserved, body brown with dorsum darker. Dense melanophores covering dorsal half of body above lateral line; loosely arranged melanophores surrounding the lateral-line scales, leaving a row of indistinct pale dots along lateral line, and extending to lower half of abdomen in front of pelvic fin, leaving a pale space below; a broad, dense band of melanophores along ventral margin of abdomen; loosely arranged melanophores on lower half of body behind VFO, gradually denser in arrangement posteriorly. Head, snout and isthmus unevenly covered with melanophores, leaving a pale space on gill cover. Pectoral fin pale with upper rays covered with scattered melanophores. Pelvic-fin rays lightly covered with melanophores. Dorsal, anal and caudal fins densely covered with melanophores. Mouth cavity and gill chamber pale, without melanophores. Abdominal cavity with black peritoneal membrane, except for lower 1/3 of the cavity, which is uniformly pale and lacking melanophores. Fresh coloration unknown but assumed similar to that of preserved condition.

***Size*.** The holotype, 186 mm SL, is a female with small ovaries and small eggs. It may be near mature size, which would indicate this may be a small species compared with congeners.

##### Etymology.

Named for the type locality, Gulf of Aden in the Western Indian Ocean.

##### Comparisons.

*Stemonosudisadenensis* sp. nov. can be distinguished by having 95 total vertebrae, which is fewer than most congeners (98–121, usually > 100; see Table 3). Among the species with < 95 total vertebrae (Table 3, lines 1–6), *S.adenensis* can be differentiated from the three species in the *S.rothschildi* complex, viz. *S.multifasciata*; *S.retrodorsalis*; and *S.rothschildi*, by its uniformly brown body, lacking blotches (vs. uneven pigmentation with distinct blotches on the dorsal and ventral margins) and a relatively anterior placement of the dorsal fin (PDV 46, vs. 49–58). For more detailed information on the species complex, see Ho et al. (2019b).

*Stemonosudisadenensis* sp. nov. differs from *S.bullisi* Rofen, 1963, in having a higher number of total vertebrae (95, vs. 84); placement of the pelvic fin closer to the dorsal fin than to the pectoral fin (prepelvic length 57.0% SL), compared to the pelvic fin being at about the midpoint between the pectoral and anal fins (prepelvic length 48.1% SL) in *S.bullisi*; and the nostrils slightly before a vertical through posterior end of maxilla (vs. well before in *S.bullisi*). It differs from *S.macrura* Ege, 1933 in having a uniformly brown dorsum (vs. two distinct rows of large melanophores on the dorsum in *S.macrura*); a distinctly greater number of prehaemal vertebrae (42 vs. 29–33); and nostrils slightly before a vertical through posterior end of maxilla (vs. well before).

In addition, *S.adenensis* sp. nov. is also similar to *Dolichosudisfuliginosa* Post, 1969, which has a uniformly black body. However, *S.adenensis* sp. nov. can be distinguished from *D.fuliginosa* by having fewer total vertebrae (95, vs. 100–104 in *D.fuliginosa*), a relatively deep body (body depth 23.5% HL, vs. 19–22% HL), and nostrils positioned slightly before the posterior end of the maxilla (vs. nostrils above the end of the maxilla).

##### Remarks.

Although described from only a single specimen, collected 38 years ago, the holotype is in good condition, and its diagnostic characters are distinctive. Despite examining many specimens in collections from around the world and consulting the results of recent surveys in the Western Indian Ocean (S. Bogorodsky, pers. comm.), we found no other specimens exhibiting the same characteristics as *S.adenensis*. Because of the current hostilities in the Gulf of Aden, it is unlikely that any surveys will be conducted in the type locality in the near future; therefore, we do not hesitate to describe this specimen as new.

#### ﻿ Stemonosudis siliquiventer

Taxon classificationAnimaliaAulopiformesParalepididae

Post, 1970

4E4FD736-AB6C-5970-AC6F-344BA31EE08B

**Figure 2.**
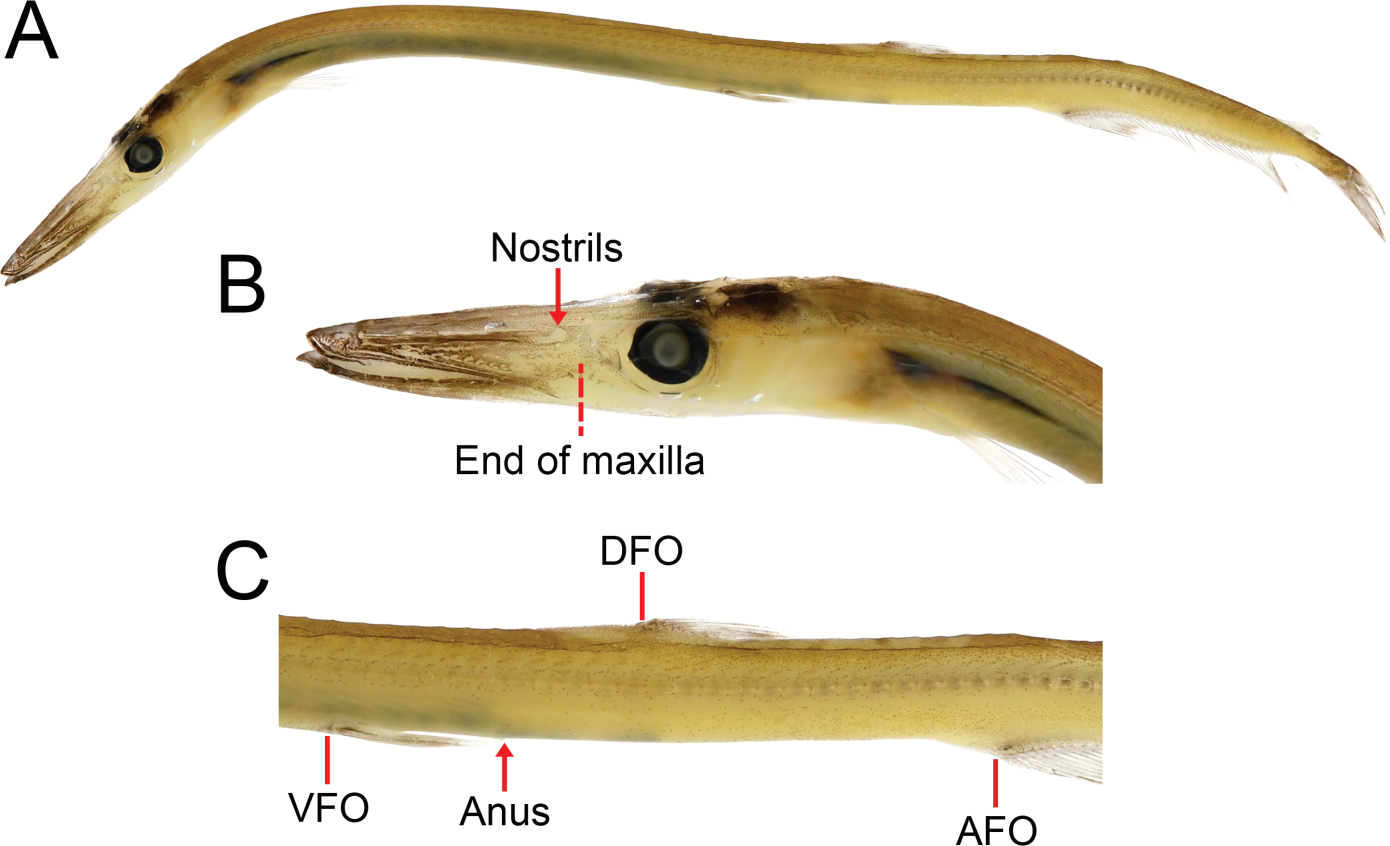
*Stemonosudissiliquiventer* Post, 1970, USNM 438714, 159 mm SL**A** lateral view **B** lateral view of trunk, arrow points to the anus and bars indicate origins of pelvic fin (VFO), dorsal fin (DFO) and anal fin (AFO).

**Figure 3.**
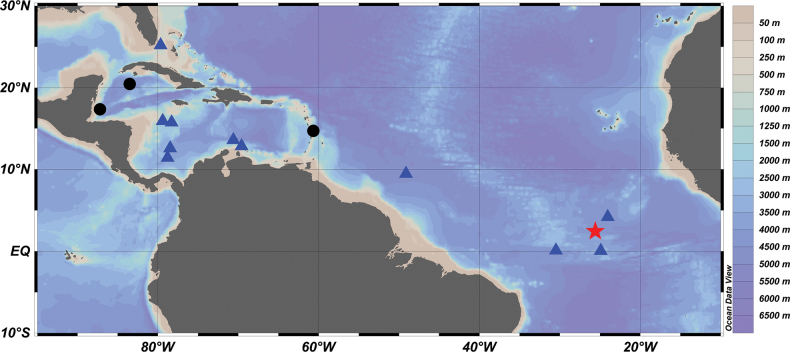
Distribution map of *Stemonosudissiliquiventer* Post, 1970 in the western central Atlantic. Star: holotype, circles: from Post (1970), and triangles: this study.

Figs 2 , 3 , Tables 1 , 2 , 3

Stemonosudis siliquiventer Post, 1970: 205, figs 1–5 (Atlantic, 3°00'S, 26°16'W, depth 2000 meters). Fukui and Ozawa 2004:293 (listed). Ho et al. 2019a:17 (Taiwan, redescription of adults). Eduardo et al. 2022:7 (Brazil, 1 specimen).

##### Original description.

Post (1970) described this species based on the holotype (159.5 mm SL), 5 paratypes (25.6–105.5 mm SL), and 7 non-types (58+–93.5 mm SL). These specimens were collected from a broad area around the central western Atlantic Ocean (Fig. 3). Except for two specimens (Table 1), all other specimens are juveniles smaller than 100 mm SL.

**Table 1.** Morphometric data for *Stemonosudisadenensis* sp. nov. and *S.siliquiventer.* Mean values are provided in parentheses.

	*S.adenensis* sp. nov.		*S.siliquiventer*			
	USNM 306145		This study		Post (1970)	
	Holotype		Non-types		Holotype	Paratype
SL (mm)	186		152–159 (n = 5)		159.5	105.5
	**In % SL**	**In % HL**	**In % SL**	**In % HL**	**In % SL**	
Head length (HL)	24.1	-	19.8–21.5 (20.7)	-	19.6	18.1
Body depth	5.7	23.7	4.0–5.2 (4.7)	19.3–26.1 (22.6)	-	-
Predorsal length	65.6	-	66.7–68.0 (67.1)	-	65.9	66.6
Prepelvic length	57.0	-	55.5–58.7 (56.7)	-	56.2	55.9
Preanus	63.0	-	60.4–63.7 (62.0)	-	61.5	61.9
Preanal length	78.0	-	77.5–82.0 (79.2)	-	78.4	79.6
V–D	8.6	35.7	9.3–11.5 (10.4)	43.5–54.2 (50.1)	9.7	10.7
V–A	21.0	87.1	22.0–23.3 (22.6)	104.2–111.1 (108.5)	22.2	23.7
VFO–anus	6.0	25.0	4.4–6.5 (5.3)	22.2–30.5 (25.3)	5.3	6.0
Eye diameter	3.8	15.6	3.1–3.5 (3.3)	14.6–17.1 (16.1)	2.9	2.3
Interorbital width	1.8	7.4	1.4–1.7 (1.6)	7.3–8.0 (7.7)	1.6	1.9
Snout length	12.2	50.7	11.2–11.9 (11.5)	53.8–56.8 (55.6)	8.7	7.9
Head depth	5.4	22.3	4.6–5.1 (4.8)	21.4–25.7 (23.0)	-	-
Head width	3.8	15.6	3.0–3.2 (3.1)	14.6–15.4 (14.9)	-	-
Pre-nostril length	9.6	40.0	9.1–9.9 (9.5)	44.3–47.3 (45.7)	-	-
Upper-jaw length	10.1	41.7	9.7–10.4 (10.0)	47.1–49.5 (48.4)	-	-
Lower-jaw length	14.2	58.9	12.8–13.9 (13.5)	61.5–68.3 (65.2)	-	-
Pectoral-fin length	8.3	34.6	9.7–10.3 (9.9)	45.8–50.2 (48.1)	-	-
Pelvic-fin length	5.4	22.3	5.1–5.8 (5.5)	24.0–29.2 (26.3)	-	-
Anal-fin base	17.4	72.1	16.1–17.2 (16.7)	78.0–84.2 (80.5)	-	-
Dorsal-fin base	3.5	14.5	3.0–3.8 (3.2)	14.0–18.3 (15.5)	-	-
Caudal-peduncle depth	1.6	6.5	1.3–1.5 (1.4)	6.3–7.3 (7.0)	-	-
Caudal-peduncle length	4.6	19.0	3.8–4.6 (4.3)	18.3–21.6 (20.6)	-	-
**In % V–A**						
V–D	41.0		40.0–52.0 (46.3)		43.7	45.1

The following diagnosis is translated directly from the original description in German (Post 1970), however, based on our observation, most of the characters are general for all species of *Stemonosudis*: “Body elongate, slender, higher than wide; ventral muscular carina present between pectoral and pelvic fins; head short, more than 5 times in SL; snout ca. 1/2 of head length; anterior edge of nostril at one eye diameter in front of eye; nostrils above posterior edge of premaxilla; eye moderately large, ca. 1/2 of head depth; lower jaw hinged under center of the eye, with a non-ossified process at the tip; fins not especially elongate; pelvic and dorsal fins behind middle of body, dorsal fin behind pelvic fin; dorsal-fin base over the middle of space between V–A; post-dorsal length ca. 1/3 of SL; anus at middle between V–D; adipose fin above last anal rays; ventral adipose fin behind anus and ended well in front of anal fin; maximum height of ventral adipose fin ca. 1/6 of that of body; body color light, transparent when fresh; band of small melanophores on dorsum (100–200µ in diameter); 23–24 clearly separated, black, peritoneal sections; lateral line extends over half of anal-fin base; lateral-line scales with two large pores; ratio of height to length of lateral-line scales 7:9”.

**Table 2.** Meristic data for *S.adenensis* sp. nov. and *S.siliquiventer.*

	*S.adenensis* sp. nov.	*S.siliquiventer*	
	USNM 306145	This study	Post (1970)
	Holotype	Non-types (n = 5)	Types
Dorsal-fin rays	10	10	10
Pectoral-fin rays	13	11–12	12
Pelvic-fin rays	9	9	9
Anal-fin rays	35	36–38	36–38
Lateral-line scales			
PVLL	39	41–43	-
PDLL	48	51–55	-
PALL	59	65–69	-
Total	81	89–97	86–87
Gill rakers			
GRI	13	9	-
GRII	27	21–23	-
GRIII	28	11–25	-
Total	68	41–58	-
Vertebrae			
PVV	38	41–42	-
PHV	42	45–46	45–46
PDV	46	52–53	-
PAV	59	65–66	-
CV	53	58–60	-
TV	95	103–105	102–103

##### Material examined.

• MCZ 39460 (3, 150–157), 21°50'50.8"N, 84°36'44.5"W, Cuba, Caribbean Sea, near surface, 26 Mar. 1939. • USNM 407802 (158), R/V Miguel Oliver, 17°42'15.8"N, 87°52'49.8"W, Belize, Caribbean Sea, 31 Jan. 2011. • USNM 438714 (159 mm SL), 15°33'17.6"N, 61°27'53.6"W, Windward Islands, Dominica, Caribbean Sea, 8 Mar. 2016, coll. L. A. Weigt & T. Christiaan.

##### Diagnosis.

A species of *Stemonosudis* distinguished by the combination of characters: a rather slender body, body depth 19–25 times in SL; DFO behind tip of pelvic fin and around middle of V–A, V–D 40.0–52.0% V–A; anus above tip of appressed pelvic fin; lateral-line scales: PVLL 41–43, PDLL 51–55, PALL 81–84, TLL 85–97; vertebrae: PVV 41–42, PHV 45–46, PDV 52–53, PAV 65–66, CV 58–60, TV 103–105; and body dark dorsally and pale ventrally without blotches.

**Table 3.** Selected data or character status of *Stemonosudis* species, divided into three subgroups with different total vertebrae. Abbreviations: D: dorsal-fin rays; A: anal-fin rays; PS: peritoneal sections; TLL: total lateral-line scales; PHV: prehaemal vertebrae; TV: total vertebrae; V: pelvic fin. Data sources: (1) this study; (2) Rofen (1966); (3) Ege (1957); (4) Ho et al. (2019b); (5) Post (1970); (6) Marshall (1955); and (7) Ho and Huang (2022).

Taxon	D	A	PS	TLL	PHV	TV	Anus/V tip	Nostrils/End of maxilla	Sources
*S.adenensis* **sp. nov.**	10	35	-	81	42	95	Above	Slightly before	1
*S.bullisi*	9	41	8	68-72	-	84	Above	Well before	2
*S.macrura*	9	33–38	12–16	-	29–33	85–95	Above	Well before	3
*S.multifasciata*	8–9	32–35	ca. 10	75–79	44–45	93–95	Above	Above	4
*S.retrodorsalis*	9–10	32–34	11	75–77	42–43	89–91	Above	Slightly before	4
*S.rothschildi*	9	32–34	9–11	74–84	42–44	89–92	Above	Slightly before	4
*S.distans*	-	ca.34	17	-	-	-	Behind	N/A	2, 3
*S.elegans*	11	36–39	7	-	44	102–104	Behind	Slightly before	2, 3
*S.gracilis*	10–11	36–39	14–15	-	43–47	98–106	Behind	Above	2, 3
*S.miscella*	11	37–38	9–11	-	42	98–101	Above	Slightly behind	2, 3
*S.siliquiventer*	10	36–38	23–26	86–97	45–46	103–105	Above	Slightly before	1, 5
*S.molesta*	12	30	7	-	-	101	Behind	Well before	6
*S.similis*	10	40	11	-	-	108?	Behind	N/A	2, 3
*S.elongata*	9–11	49–51	31–32	102–108	48–49	115–121	Behind	Slightly behind	3, 7
*S.intermedia*	9–11	43–48	16–18	86–92	49; 53–56	111–113; 116–121	Above	Slightly behind	2, 3

##### Description

**(based on 5 specimens examined herein).** Body relatively slender as in most *Stemonosudis* species; strongly compressed. Caudal peduncle moderately long, its length about 1.1–1.5 times eye diameter. Ventral adipose fin weakly developed, with a narrow transparent membrane along ventral abdominal ridge in front of pelvic fin and well-developed along ventral margin between anus and anal fin. Anus located approximately above tip of the appressed pelvic fin, about midpoint of V–D.

Head relatively long, its length 4.7–5.0 in SL; snout long and pointed, 1.8–1.9 in HL and 8.4–8.9 in SL. Mouth terminal, moderately large, its gape extending to slightly more than one eye diameter before eye with mouth opened; tip of lower jaw slightly upturned, with a small fleshy knob. Eye moderately small. Interorbital space rather narrow, 12.4–13.7 in HL; some straight symmetric ridges on top of head and snout. Posterior end of maxilla extending to about 1/2 eye diameter before the eye. A skin fold originating from about middle of snout and ending near a vertical through anterior margin of eye pupil. Two nostrils at or slightly before a vertical through posterior end of maxilla, situated about 3/5 eye diameter in front of eye. Numerous sensory canals on snout, cheek, operculum, and jaws; numerous sensory pores on dorsal surface of snout; irregular rows of small pores on under surface of lower jaw.

Filaments present on all four gill arches. Fourth arch connected almost entirely to the gill cavity wall by membranes. Pseudobranchs present, anterior half inside a deep pocket.

Dorsal fin small with a short base. DFO well behind pelvic fin and middle of the fish, at about middle of V–A or slightly before, V–D 40.0–52.0% V–A. Pectoral fin slender, upper rays distinctly longer than lower rays; its base slightly behind a vertical through posterior margin of gill cover, upper base tangent to a horizontal though lower margin of eye. A small fleshy pocket behind pectoral-fin base. Pelvic fin slightly behind middle of the fish; a small, slender axial scale behind pelvic fin base. Anal fin with a short base, originating about four-fifths along the fish. Adipose fin relatively large, its base about same as eye diameter, situated above rear portion of anal-fin base.

Two or 3 small fangs at tip of upper jaw, followed by single row of numerous (>80) small retrorse teeth along upper jaw, becoming gradually smaller posteriorly. Vomerine teeth absent. Two or 3 depressible or fixed fangs at front of lower jaw, followed by two rows of fangs on lower jaw forming about 7 or 8 tooth pairs, those in inner row distinctly longer and depressible, with knife-like tips; those in outer row much shorter, fixed and retrorse. Two rows of fangs on each palatine, anterior teeth forming 5–9 widely-spaced tooth pairs, those in inner row depressible and distinctly longer than those of outer row, which are small and fixed; posterior portion with single row of 6–8 small fixed teeth. Two long rows of small retrorse teeth on tongue.

Gill rakers present on all gill arches, assumed not fully developed in specimens examined; small, shield-shaped, mostly with 1–3 small teeth and a narrow base, teeth not especially emergent over margin of gill arch. Teeth on pharyngeal arch slender, forming an oval patch with approximately 4 rows at middle. Single row of small teeth on each fifth ceratobranchial, both forming a V-shaped pattern.

Body scaleless, except for a single row of lateral-line scales originating above pectoral girdle and extending to about 2/3 of anal-fin base. Lateral-line scales relatively long, longer than high, becoming gradually smaller and narrower posteriorly; row of 2 (few with 3) pores on upper and lower margin of each scale, anterior pore larger; and pore on broader of each scale smaller. Luminescent duct absent.

***Coloration*.** Body light gray, with unevenly distributed melanophores; large unpigmented areas on gill cover, abdominal region and body axis; dense melanophores on most surfaces of snout, both jaws and dorsal surface of head; dorsum densely covered with melanophores, these extending downward to lateral line; abdominal ridge with loosely arranged melanophores; lower half of body with scattered melanophores behind anus, these becoming gradually more dense posteriorly, forming a darker caudal peduncle; pectoral fin covered with few melanophores; other fins densely covered by melanophores. Gill chamber, gill arches and filaments unpigmented. Peritoneal sections 23–26.

##### Remarks.

Table 1 provides the morphometric data for our specimens compared to the two largest types (159.5 mm SL holotype and 105.5 mm SL paratype) mentioned in the original description. The morphometric and meristic values are generally consistent between these types and our specimens, with some exceptions as noted below. Our specimens have nostrils slightly anterior to the posterior end of the maxilla, whereas the holotype has nostrils positioned directly above the end of the maxilla (as observed in the original drawing; Post 1970). The snout length is shorter in the two types (8.7% and 7.9% SL, respectively) compared to our specimens (11.2–11.9% SL). The eye diameter is slightly larger in our specimens (3.1–3.5% SL), while the eyes are smaller in the two types (2.9% and 2.3% SL, respectively). Additionally, our specimens exhibit a greater number of total lateral-line scales (89–97) compared to the two types (86–87), which is within the variation range of *Stemonosudis* species.

In the Atlantic Ocean, only four *Stemonosudis* species were recorded. *Stemonosudissiliquiventer* differs from *S.bullisi* Rofen, 1962 in having origins of dorsal and pelvic fins well behind middle of space between pectoral-fin base to anal-fin base (vs. at about middle of the space); from *S.intermedia* in having 103–105 total vertebrae (vs. 111–121); and from *S.gracilis* (Ege, 1933) in lacking blotches on dorsum and ventral margin (vs. 3 or 4 blotches on dorsum and 2 or 3 on ventral margin) and 23–26 peritoneal sections (vs. 14–15).

Moreover, in the Indo-Pacific region, *S.siliquiventer* is different from *S.elegans* (Ege, 1933) (Indo-west Pacific) and *S.miscella* (Ege, 1933) (western Pacific), *S.multifasciata* Ho, Russell, Graham & Psomadakis, 2019 (Indo-west Pacific), *S.rothschildi* Richards, 1967 (circumglobal), *S.retrodorsalis* Ho, Russell, Graham & Psomadakis, 2019 (Indo-west Pacific) and *S.similis* (Ege, 1957) (western Pacific) in lacking blotches on dorsum and ventral margin (vs. several distinct blotches on dorsum and ventral surface) and 23–26 peritoneal sections (vs. 3–11); from *S.molesta* (Marshall, 1955) (New Zealand) in having 10 dorsal-fin rays (vs. 13) and 36–38 anal-fin rays (vs. 30); from *S.distans* (Ege, 1957) (western Pacific) in having 36–38 anal-fin rays (vs. 34) and 23–26 peritoneal sections (vs. 17); from *S.macurus* (Ege, 1933) (Indo-Pacific) in lacking distinct rows of large melanophores on dorsum (vs. two distinct rows of large melanophores on dorsum); and from *S.elongata* (Ege, 1933) by having 36–38 anal-fin rays (vs. 48–51).

## Supplementary Material

XML Treatment for Stemonosudis adenensis

XML Treatment for Stemonosudis siliquiventer

## ﻿Additional information

### Author ORCIDs

Hsuan-Ching Ho https://orcid.org/0000-0003-1154-601X

Tsung-Yu Yang https://orcid.org/0000-0003-4302-120X

### Data availability

All of the data that support the findings of this study are available in the main text.
